# NDM-63: a novel NDM metallo-β-lactamase variant in the L3 loop, from a *Klebsiella pneumoniae* clinical isolate

**DOI:** 10.1128/aac.01286-25

**Published:** 2025-12-29

**Authors:** Selene Rebecca Boncompagni, Alberto Antonelli, Benedetta Casciato, Filippo Pieralli, Alejandro J. Vila, Diego M. Moreno, Tommaso Giani, Gian Maria Rossolini

**Affiliations:** 1 Department of Experimental and Clinical Medicine, University of Florence 9300, Florence, Italy; 2 Microbiology and Virology Unit, Florence Careggi University Hospital 18561 , Florence, Italy; 3 Emergency and Internal Medicine Department, Careggi University Hospital 18561 , Florence, Italy; 4 Facultad de Ciencias Bioquímicas y Farmacéuticas, Universidad Nacional de Rosario 28237, Rosario, Argentina; 5 CWRU-Cleveland VAMC Center for Antimicrobial Resistance and Epidemiology (Case VA CARES) 2546, Cleveland, Ohio, USA; 6 CONICET, Universidad Nacional de Rosario, Instituto de Biología Molecular y Celular de Rosario (IBR) 28237, Rosario, Argentina; 7 CONICET, Universidad Nacional de Rosario, Instituto de Química Rosario (IQUIR) 28237, Rosario, Argentina; University of Fribourg, Fribourg, Switzerland

**Keywords:** carbapenemase, New-Delhi metallo-β-lactamase, enzyme variant, *Klebsiella pneumoniae*

## Abstract

NDM-type metallo-β-lactamases (MBLs) are among the most widespread acquired carbapenemases in carbapenem-resistant Enterobacterales. As with other β-lactamases, allelic variability occurs among NDM-type MBLs, with almost 100 variants so far reported, differing by single or multiple amino acid substitutions or insertions, which may have implications for enzymatic activity. In this study, we report on a novel NDM variant, NDM-63, identified in a carbapenem-resistant ST147 *Klebsiella pneumoniae* from a surveillance rectal swab. Compared to NDM-1, NDM-63 features an original array of changes in the L3 loop, including deletion of phenylalanine at position 70 and two amino acid substitutions (G69S and A72H), due to a four-nucleotide deletion plus a nucleotide insertion in the gene region encoding the L3 loop. When expressed in *Escherichia coli* under isogenic conditions, NDM-63 conferred a resistance profile overall similar to NDM-1, but exhibiting a lower level of resistance to carbapenems and cefepime, while remaining susceptible to inhibition by taniborbactam. Present findings expand current knowledge on the structural plasticity of NDM-type MBLs and highlight that variability in the L3 loop, which contributes to delimitation of the active site, could also tolerate amino acid deletions without loss of enzymatic activity. A virtually identical *K. pneumoniae* carrying a non-functional *bla*_NDM_ allele entailing only the nucleotide insertion observed in *bla*_NDM-63_ (which might have played a role in the evolution of *bla*_NDM_) was also isolated from a bloodstream infection that occurred in the same patient, yielding a misleading result of molecular diagnostic testing due to the lack of enzyme activity despite the presence of the target gene.

## INTRODUCTION

The prevalence of carbapenemase-producing Enterobacterales (CPE) has increased since the early 2000s, representing a major health threat (1). Among CPE, strains producing metallo-β-lactamases (MBLs) are those posing a major challenge due to the very limited treatment options (2).

On a global scale, NDM-type enzymes stand out as the most widespread acquired MBLs among CPE (3). Their epidemiological success is dependent on the ability for horizontal dissemination with propensity to be associated with successful clonal lineages of Enterobacterales (4), and on the lipidated nature of the enzyme, which improves stability even in low-zinc environments such as infected tissues (5).

As with other β-lactamases, allelic variability exists among NDM-type MBLs. In fact, since the initial report of NDM-1 (6), almost 100 variants which differ by single or multiple amino acid substitutions or insertions have been described (https://www.ncbi.nlm.nih.gov/pathogens/refgene/#ndm).

In this work, we describe a new NDM variant, NDM-63, which carries an original sequence modification in the L3 loop, detected in a carbapenem-resistant *Klebsiella pneumoniae* of clinical origin. We also describe a non-functional *bla*_NDM_ allele, detected in a *K. pneumoniae* isolated from the same patient, which caused an invasive infection and posed challenges with molecular diagnostic testing.

## RESULTS AND DISCUSSION

In 2023, an elderly patient was admitted to the Emergency Room of our hospital due to a high-grade unremitting fever and was subsequently moved to the intensive-care observation unit. Blood cultures collected at admission tested positive for gram-negative bacilli upon microscopic observation. Testing of the positive blood culture with a molecular syndromic panel yielded positivity for *K. pneumoniae* group plus *bla*_NDM_ and *bla*_CTX-M_ resistance genes. Following subculture, the isolate was identified by MALDI-ToF as *K. pneumoniae* (Kp_25151). Unexpectedly, antimicrobial susceptibility testing (AST) showed that Kp_25151 was susceptible to carbapenems, piperacillin-tazobactam, ceftazidime-avibactam, ceftolozane-tazobactam, and meropenem-vaborbactam, but resistant to third-generation and fourth-generation cephalosporins and aztreonam. Molecular analysis by RT-PCR confirmed positivity for the *bla*_NDM_ and *bla*_CTX_ targets, while lateral-flow immunochromatography assay (LFIA) testing was negative for NDM production (Table 1).

**TABLE 1:** Antimicrobial susceptibility of *K. pneumoniae* Kp_25151 and Kp_25048, *E. coli* DH10B (pBC/NDM-63), *E. coli* DH10B (pBC/NDM-1), and *E. coli* DH10B carrying the empty vector (pBC-SK)^*a*^

Antimicrobial agent	MIC (mg/L) (category)				
	Kp_25151	Kp_25048	DH10B pBC-SK	DH10B pBC/NDM-1	DH10B pBC/NDM-63
Amoxicillin-clavulanic acid	64 (R)	>64 (R)	2	>1,024	>1,024
Piperacillin-tazobactam	4 (S)	64 (R)	1	>1,024	>1,024
Ceftazidime	64 (R)	>1,024 (R)	0.5	>1,024	>1,024
Ceftazidime-avibactam	1 (S)	>1,024 (R)	0.5	>1,024	>1,024
Ceftriaxone	512 (R)	512 (R)	0.06	>1,024	>1,024
Cefepime	16 (R)	64 (R)	0.06	1,024	256
Cefepime-taniborbactam	0.125 (N.A.)	1 (N.A.)	0.06	8	8
Cefiderocol	0.25 (S)	1 (S)	0.06	2	1
Ceftolozane-tazobactam	1 (I)	>1,024 (R)	0.125	>1,024	>1,024
Aztreonam	>32 (R)	>32 (R)	0.25	0.5	0.5
Aztreonam-avibactam	0.125 (S)	0.125 (S)	0.25	0.125	0.125
Ertapenem	≤0.5 (S)	64 (R)	0.002	128	32
Imipenem	0.5 (S)	16 (R)	0.125	128	32
Meropenem	0.125 (S)	8 (I)	0.06	128	32
Meropenem-vaborbactam	0.06 (S)	8 (S)	0.06	128	32
Amikacin	>16 (R)	>16 (R)	–	–	–
Gentamicin	>8 (R)	>8 (R)	–	–	–
Ciprofloxacin	>1 (R)	>1 (R)	–	–	–
Colistin	≤0.5 (S)	≤0.5 (S)	–	–	–
Tigecycline	0.5 (N.A.)	≤0.25 (N.A.)	–	–	–
Trimethoprim-sulfamethoxazole	>8 (R)	>8 (R)	–	–	–

^*a*^ Data are median values of three independent measurements. N.A., clinical breakpoints not available. –, not performed.

Interestingly, a surveillance rectal swab taken at admission also tested positive for *bla*_NDM_ and *bla*_CTX-M_ genes by RT-PCR, and culture of the rectal swab on selective chromogenic medium for carbapenem-resistant Enterobacterales (CRE) yielded a *K. pneumoniae* isolate (Kp_25048) which tested positive for *bla*_NDM_ and *bla*_CTX-M_ by RT-PCR, and for NDM production by LFIA. AST of Kp_25048 showed a broad-spectrum β-lactam resistance profile, including carbapenems and also the new β-lactamase inhibitor combinations (BLICs) (Table 1), consistent with NDM production.

**Fig 1:**
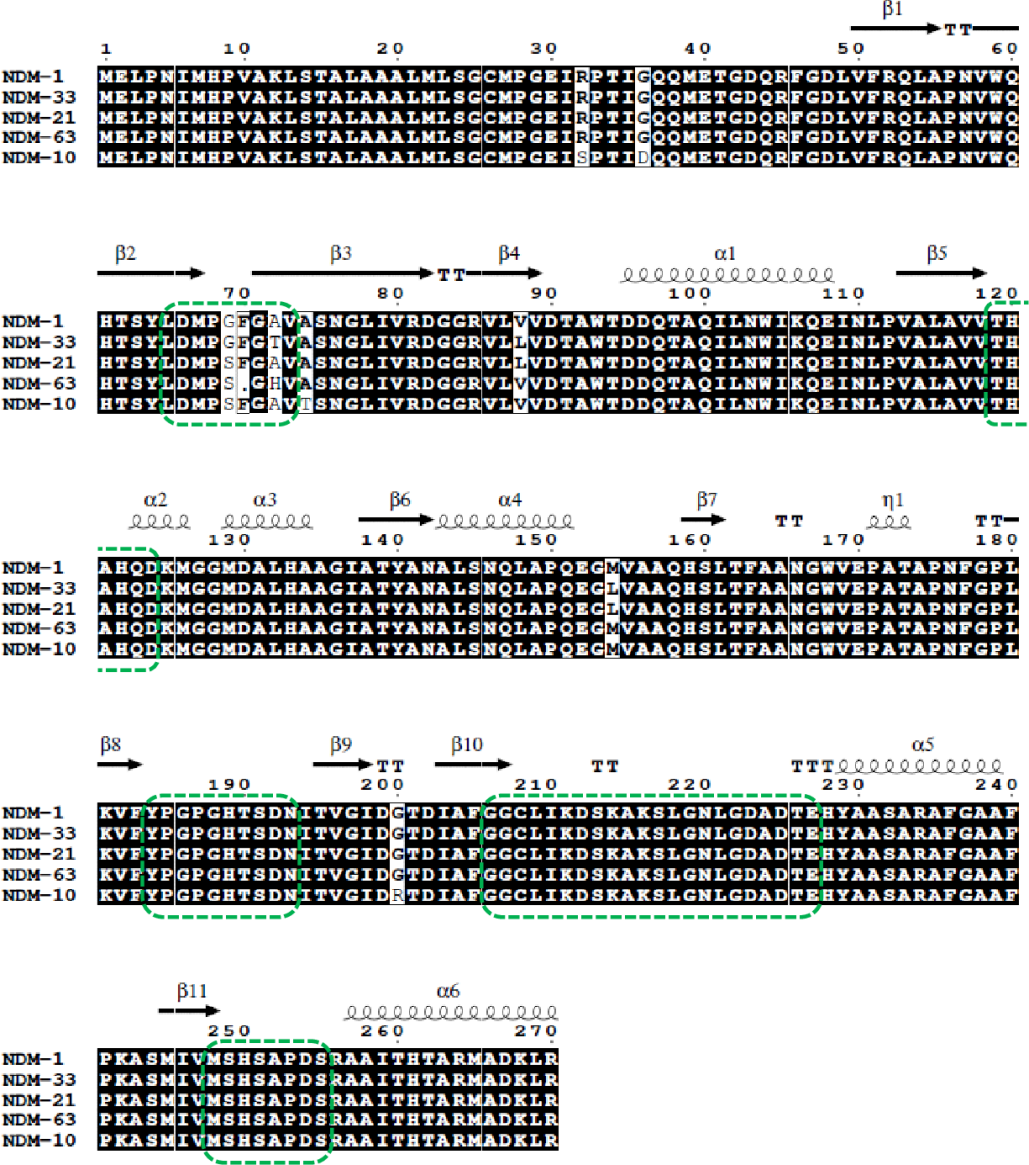
Amino acid sequence alignment of NDM-1 and its derivative variants showing an altered active-site loop between strands β2 and β3. The five active-site loops are indicated by green boxes. The location of α-helices and β-sheets is indicated above the sequences (from PDB ID: 3PG4). The conserved residues are indicated in black, while the amino acid substitutions are indicated in white. The figure was generated using ESPript (http://espript.ibcp.fr/ESPript/ESPript/).

WGS analysis with a combined short-read and long-read approach revealed that Kp_25151 and Kp_25048 had identical core genomes and belonged to sequence type (ST)147, with KL64 (*wzi*64) capsular locus and O2v1 locus, being members of the dominant lineage associated with the large outbreak of NDM-producing *K. pneumoniae* ongoing in Tuscany since 2019 (7). Moreover, the two genomes shared an identical virulome comprising genes for siderophores (yersiniabactin, aerobactin), iron metabolism proteins, and regulators of the mucoid phenotype; an identical plasmidome including IncR, IncFIB(pQil)_1, HIB-FIB(Mar), and ColE-like plasmids; and a complex resistome to multiple classes of antimicrobials (aminoglycosides, sulfonamides, trimethoprim, rifampicin, macrolides, phenicols, and β-lactams), which was identical except for the *bla*_NDM_ genes (Table S1). In particular, Kp_25151 carried a *bla*_NDM_ gene which, compared with *bla*_NDM-1_ (accession number: NG_049326.1), exhibited a single-nucleotide (T) insertion between positions 215 and 216, resulting in a frameshift followed by a premature stop codon. This explained the lack of production of a functional NDM enzyme and the carbapenem-susceptible phenotype of Kp_25151, despite the positivity for *bla*_NDM_ returned by molecular testing. Conversely, Kp_25048 carried a *bla*_NDM_ gene exhibiting the same nucleotide insertion observed in Kp_25151 but also a four-nucleotide deletion (ΔGGTT) at positions 205–208. Altogether, the combined alterations resulted in suppression of the frameshift caused by the single-nucleotide insertion plus some amino acid modifications in the L3 loop, including deletion of phenylalanine at position 70 and substitution of two amino acids (G69S and A72H) in comparison with NDM-1 (numbering referred to NCBI Reference Sequence WP_004201164.1), yielding an original NDM variant that was named NDM-63 (Fig. 1).

In both Kp_25151 and Kp_25048, the *bla*_NDM_ genes were located on identical genetic platforms, represented by a 46 kb IncFIB(pQil) plasmid, which showed 99.9% identity (100% coverage) with the pKP51_NDM1 plasmid from an ST147 *K. pneumoniae* from the same region (8). Since these plasmids are lacking conjugal transfer genes, they are expected to not be self-transferable by conjugation (7).

NDM-1, the first described NDM-type MBL, is composed of 270 amino acids (6). Since its discovery in 2009 (6), at least 89 allelic variants have been described (NCBI National Database of Antibiotic Resistant Organisms, last accessed on 12 November 2025), with amino acid substitutions observed at 67 of the 270 protein positions (Table S2). Typically, NDM variants exhibit between 1 and 6 amino acid substitutions when compared to NDM-1, the most prevalent being M154L and V88L, observed in 50 and 39 NDM variants, respectively. M154L was shown to increase the zinc binding affinity (9), while V88L in combination with M154L (e.g., in NDM-5) was shown to increase the protein stability against proteases (10). NDM-18 is the sole exception showcasing an insertion (a tandem repeat of four amino acids, QRFG, from positions 44 to 48 of NDM-1) (Table S2). Compared to NDM-1 and other reported variants, NDM-63 exhibited an original array of sequence alterations, including a G69S substitution (previously reported in NDM-10 and NDM-21, which also presented additional substitutions and exhibited a functional behavior similar to that of NDM-1 and NDM-5, respectively) (11, 12), an original A72H substitution, and an original deletion of the F70 residue, which so far appears to be the first amino acid deletion observed in an NDM variant (Fig. 1).

To evaluate the potential functional significance of the alterations present in NDM-63, we tested susceptibility to a representative panel of β-lactams and BLICs of *Escherichia coli* DH10B engineered to produce NDM-63 and NDM-1, under otherwise isogenic conditions. Production of NDM-63 in *E. coli* DH10B (pBC/NDM-63) conferred a phenotype overall similar to that of NDM-1, although lower MIC values were observed with carbapenems and cefepime, suggesting that the alterations present in the L3 loop of NDM-63 could be of some functional significance, albeit not altering the overall profile. Moreover, taniborbactam was able to reduce cefepime MICs of strains producing NDM-63, suggesting that this variant retains susceptibility to that inhibitor, which is affected with NDM-9, NDM-30, and NDM-60 (13–15) (Table 1).

To better illustrate the structural differences between NDM-1 and NDM-63, we built an *in silico* model of NDM-63 and subjected it to 500 ns molecular dynamics (MD) simulations. Figure S2 shows representative snapshots of NDM-1 and NDM-63 extracted from the simulation trajectories, highlighting the G69S and A72H substitutions and the deletion of F70. These alterations are located in the L3 loop, a flexible region adjacent to the active site that acts as a mobile flap modulating substrate access and binding. Although not directly involved in catalysis, the observed changes are consistent with the functional results obtained from isogenic strain experiments. Further studies will be necessary to clarify the impact of the amino acid substitutions observed in NDM-63 on the enzyme’s functional profile and stability.

In conclusion, this observation emphasizes the structural variability that can be accommodated by NDM-type MBLs by describing a new variant with an original array of sequence alterations in the L3 loop of the enzyme, which is a flexible loop known to act as a mobile flap that modulates substrate access, binding, and stabilization of reaction intermediates (16). The alterations present in NDM-63, including an amino acid deletion, are compatible with enzyme function, which retains an MBL-type profile, although they apparently could entail functional relevance. Further characterization of this enzyme could be of interest in providing additional insights into the structure-function relationships of NDM-type MBLs, which so far appear to be the most successful acquired MBLs spreading among Enterobacterales in the clinical setting.

Interestingly, while Kp_25048 (carrying *bla*_NDM-63_) was found as a colonizer, a virtually identical strain carrying an inactivated *bla*_NDM_ variant apparently related to *bla*_NDM-63_ (e.g., carrying the same T insertion at position 217, but not the four-nucleotide deletion which suppresses the frameshift) was isolated from a bloodstream infection occurring in the same patient. This resulted in misleading information about the expected resistance profile of the infecting pathogen when using molecular testing of the positive blood culture, due to the lack of enzyme activity despite the presence of the target gene, underscoring the potential limitations of contemporary molecular syndromic platforms due to the emergence of allelic variants of carbapenemase genes. The similarity between the two strains and the nature of the alterations found in the two *bla*_NDM_ alleles also led us to speculate that the inactivated *bla*_NDM_ allele might have played a role in the evolution of *bla*_NDM-63_.

## MATERIALS AND METHODS

### Clinical isolates, antimicrobial susceptibility testing, and preliminary detection of resistance mechanisms

*K. pneumoniae* Kp_25151 and Kp_25048 were isolated from blood culture and a rectal swab, respectively, taken from a patient admitted to the Emergency Room at Florence Careggi University Hospital (Tuscany, Italy). Identification of clinical isolates was initially carried out by MALDI-ToF mass spectrometry (Bruker MS system, Bruker Daltonics, Germany) and subsequently confirmed by WGS results (see below). For molecular testing of positive blood culture, the BCID syndromic panel (bioMèrieux, Marcy l’Etoile, France) was used. AST was performed by broth microdilution (BMD) using a commercial plate (ITGN E1-184-100 panel, Merlin Diagnostika GmbH, Bornheim, Germany), in accordance with the manufacturer’s instructions. Antimicrobial agents not included in the commercial panel were tested by reference BMD (17) using powders from commercial sources (avibactam from Aobious [Gloucester, MA, USA]; imipenem, meropenem, relebactam, and vaborbactam from Merck [Rahway, NJ, USA]; aztreonam from United Biotech [Delhi, India]; and taniborbactam from MedChem Express [Monmouth Junction, NJ, USA]). AST of BLICs was performed using fixed concentrations of inhibitors (avibactam, relebactam, and taniborbactam at 4 mg/L; vaborbactam at 8 mg/L). Susceptibility to cefiderocol (Merck Life Science, Rahway, NJ, USA) was evaluated by reference BMD using iron-depleted medium (18, 19). AST data were obtained from three independent measurements. *K. pneumoniae* ATCC BAA-2814, *Pseudomonas aeruginosa* ATCC 27853, and *E. coli* ATCC 25922 were used as quality control strains. Susceptibility interpretations were based on clinical breakpoints from the European Committee on Antimicrobial Susceptibility Testing (v. 15.0 2025, available at https://www.eucast.org/clinical_breakpoints). Carbapenemase gene detection in surveillance swabs and bacterial isolates was performed using the Allplex Entero-DR molecular assay (Seegene, South Korea). Carbapenemase production in bacterial isolates was evaluated by the RESIST-5 O.O.K.N.V. lateral-flow immunochromatography assay (LFIA) (Coris BioConcept, Belgium).

### Genomic analysis

Total genomic DNA was extracted from bacterial cultures grown on Mueller-Hinton agar plates (Oxoid, Basingstoke, UK) using the Qiagen DNeasy PowerLyzer PowerSoil Kit (Qiagen, Hilden, Germany) and subjected to WGS analysis using an Illumina MiSeq platform (Illumina Inc., San Diego, USA) with a paired-end approach (2 ×  250 bp) and a Nanopore MinION platform (Oxford Nanopore Technologies, Oxford, UK), as previously described (7). *De novo* assemblies were generated using Unicycler v0.5.0, and downstream sequence analyses were performed using Kleborate (https://github.com/katholt/Kleborate), ABRicate (https://github.com/tseemann/abricate), and BLAST (https://blast.ncbi.nlm.nih.gov/blast/Blast.cgi), as described previously (7). Clonal relatedness was investigated by generation of core-genome single-nucleotide polymorphisms (SNPs) using Snippy v. 4.6.0 (https://github.com/tseemann/snippy), with the complete genome of *K. pneumoniae* KP-1PI as reference (an ST147-NDM-*K. pneumoniae* isolate from the Tuscany outbreak started in 2019) (accession number: CP071027).

### Cloning and expression of *bla*_NDM_ variants

The *bla*_NDM_ genes (along with the identical natural promoters) were amplified from the genomic DNA of *K. pneumoniae* KP-1PI and Kp_25048 (for *bla*_NDM-1_ and *bla*_NDM-63_, respectively) using the primers BamHI-NDM-ext-fw (5′-CCGGATCCCGTTAGATTGGCTTACACCATTAG-3′) and HindIII-NDM-ext-rev (5′-CCGAAGCTTCATGGCATCGAGATCATCCAAC-3′) and the Q5 High-Fidelity DNA Polymerase (New England Biolabs). The PCR product was digested with BamHI and HindIII (Promega, USA) and ligated into the linearized (BamHI and HindIII digested) pBC-SK(-) vector (Agilent Technologies, Santa Clara, CA, USA). The resulting recombinant plasmids, pBC/NDM-1 and pBC/NDM-63, were transformed into electrocompetent *E. coli* strain DH10B (Invitrogen, Waltham, MA, USA) using medium supplemented with 85 mg/L of chloramphenicol for selection. The authenticity of the recombinant clones was confirmed by Sanger sequencing (Eurofins, Luxembourg City, Luxembourg) of recombinant plasmid DNA on both strands.

### Structural modeling

Representative structures of NDM-1 and NDM-63 were obtained from MD simulations performed with the Amber24 software package (https://ambermd.org/index.php). The initial coordinates for NDM-1 were taken from the crystallographic structure available in the Protein Data Bank (PDB ID: 3SPU) (16), whereas the NDM-63 model was predicted using AlphaFold3 (20). Each protein was solvated in a truncated octahedral periodic box containing explicit water molecules, ensuring a minimum distance of 9 Å between any solute atom and the box boundaries. Sodium and chloride ions were added to neutralize the system and reach an ionic strength of 0.1 M. The FF19SB force field (21) was used to describe protein atoms, and the OPC model (22) was applied for water molecules. Parameters for active-site residues were taken from previously published studies (23). The MD protocol comprised four stages: (i) energy minimization of the solvated system, (ii) heating for 10 ps from 0 to 300 K under constant volume, (iii) equilibration for 20 ps at 1 bar and constant pressure, and (iv) a 500 ns production run under constant temperature and pressure. Representative conformations were selected by cluster analysis performed with cpptraj (https://github.com/Amber-MD/cpptraj).

## Data Availability

Whole-genome sequencing data are accessible via BioProject PRJNA1126178.

## SUPPLEMENTAL MATERIAL

Supplemental material aac.01286-25-s0001.docx Tables S1 and S2; Fig. S1 and S2.

ASM does not own the copyrights to Supplemental Material that may be linked to, or accessed through, an article. The authors have granted ASM a non-exclusive, world-wide license to publish the Supplemental Material files. Please contact the corresponding author directly for reuse.
