# Supplementary material for: NDM-63: a novel NDM metallo-β-lactamase variant in the L3 loop, from a Klebsiella pneumoniae clinical isolate
Source: Antimicrob Agents Chemother. 2025 Dec 29;70(2):e01286-25. doi: 10.1128/aac.01286-25 (PMC12888852; doi:10.1128/aac.01286-25)

**Table S1. Genomic characteristics of Kp_25048 and Kp_25151 clinical strains.**

| **Isolate** | **Source** | **ST** | **Capsular Type** | **Serotype** | **Plasmid Types** | **Resistome** |
| --- | --- | --- | --- | --- | --- | --- |
| Kp_25048 | Rectal swab | 147 | KL64 | O2v1 | HIB-FIB(Mar), ColE-like, IncFIB(pQil), IncR | *bla*_NDM-63_, *bla*_CTX-M-15_, *bla_OXA-1_, bla*_OXA-9_, *bla*_SHV-11_, *bla*_TEM-1A_, *aac(6')-*Ib-cr, *aac(6')*-Ib, *ant(3'')*-Ia, *aph(3')*-VI, *arm*A, *cat*B3, *dfr*A5, *fos*A, *mph*(A), *mph*(E), *msr*(E), *oqxA*, *oqxB*, *sul1*, *sul2* |
| Kp_25048 | blood | 147 | KL64 | O2v1 | HIB-FIB(Mar), ColE-like, IncFIB(pQil), IncR | *bla*_NDM*_, *bla*_CTX-M-15_, *bla_OXA-1_, bla*_OXA-9_, *bla*_SHV-11_, *bla*_TEM-1A_, *aac(6')-*Ib-cr, *aac(6')*-Ib, *ant(3'')*-Ia, *aph(3')*-VI, *arm*A, *cat*B3, *dfr*A5, *fos*A, *mph*(A), *mph*(E), *msr*(E), *oqxA*, *oqxB*, *sul1*, *sul2* |
| *, interrupted |  |  |  |  |  |  |

**Table S2. Amino acid substitutions (referred to NDM-1) among NDM variants and their first identified host (NCBI National Database of Antibiotic Resistant Organisms, last accessed on July 20, 2025).**

| **NDM variant** | **GenBank protein** | **Location of Amino Acid(s) Substitution (vs NDM-1)** | | | | **Source Organism(s)** |
| --- | --- | --- | --- | --- | --- | --- |
|  |  | **A-Helices** | **β-Strands** | **Loop** | **Signal peptide** |  |
| NDM-2 | AEA41876.1 | - | - | P28A | - | *A. baumannii* |
| NDM-3 | AFK80349.1 | D95N | - | - | - | *E. coli* |
| NDM-4 | AFB82585.1 | - | - | M154L | - | *E. coli* |
| NDM-5 | AEN03071.1 | - | V88L | M154L | - | *E. coli* |
| NDM-6 | AEX08599.1 | A233V | - | - | - | *E. coli* |
| NDM-7 | AFQ31613.1 | D130N | - | M154L | - | *E. coli* |
| NDM-8 | BAM84089.1 | D130G | - | M154L | - | *E. coli* |
| NDM-9 | AGU91756.1 | - | - | E152K | - | *K. pneumoniae* |
| NDM-10 | AGT37351.1 | - | A74T | R32S, G36D, G69S, G200R | - | *K. pneumoniae* |
| NDM-11 | AJE61443.1 | - | - | M154V | - | *E. coli* |
| NDM-12 | BAO79439.1 | G222D | - | M154L | - | *E. coli* |
| NDM-13 | BAQ02518.1 | D95N | - | M154L | - | *E. coli* |
| NDM-14 | AJP18054.1 | D130G | - | - | - | *A. lwoffii* |
| NDM-15 | AKF43458.1 | A233V | - | M154L | - | *E. coli* |
| NDM-16a | AKZ20823.1 | R264H | - | - | - | *K. pneumoniae* |
| NDM-16b | BCO01847.1 | A233V | V88L | M154L | - | *E. coli* |
| NDM-17 | AOT73359.1 | - | V88L | M154L, E170K | - | *E. coli* |
| NDM-18 | APZ75411.1 | - | - | QRFGD (44-48) | - | *P. rettgeri* |
| NDM-19 | ASC49561.1 | D130N | - | M154L, A233V | - | *E. coli* |
| NDM-20 | AQY45923.1 | - | V88L | M154L, R270H | - | *E. coli* |
| NDM-21 | ATJ25942.1 | - | V88L | G69S, M154L | - | *E. coli* |
| NDM-22 | AWI33311.1 | - | - | M248L | - | *E. cloacae* |
| NDM-23 | AWU66462.1 | I101L | - | - | - | *K. pneumoniae* |
| NDM-24 | AWU66463.1 | - | V88L | - | - | *P. stuartii* |
| NDM-25 | AYF56302.1 | - | A55S | - | - | *K. pneumoniae* |
| NDM-26 | AYN72942.1 | G222S | V88L | M154L | - | *E. coli* |
| NDM-27 | AYP70146.1 | D95N, A233V | - | - | - | *E. coli* |
| NDM-28 | QAT97614.1 | A266V | - | - | - | *K. pneumoniae* |
| NDM-29 | QFZ95817.1 | D130N | - | - | - | *K. pneumoniae* |
| NDM-30 | QPK66974.1 | D223Y | - | - | - | *K. oxytoca* |
| NDM-31 | QPK66975.1 | P171T | - | - | - | *C. werkmanii* |
| NDM-33 | UBU60937.1 | - | A72T, V88L | M154L | - | *E. coli* |
| NDM-34 | QVU28093.1 | - | L49M, L54I, V88L | - | - | *V. parahaemolyticus* |
| NDM-35 | QVU28098.1 | - | G84D, V88L | M154L | - | *E. coli* |
| NDM-36 | MBT0799486.1 | A233V, H261Y | V88L | M154L | - | *E. coli* |
| NDM-37 | ULB43298.1 | H261Y | V88L | M154L | - | *E. coli* |
| NDM-38 | QWO25674.1 | - | - | M27V | - | *P. rettgeri* |
| NDM-39 | QYZ89892.1 | - | V88L | M154L | - | N.A |
| NDM-40 | QYZ89893.1 | - | - | - | P4R | *A. baumannii* |
| NDM-41 | UAX43328.1 | - | V88L | M154L | L13V | *K. pneumoniae* |
| NDM-42 | UOU25745.1 | - | - | P28L | - | *A. baumannii* |
| NDM-43 | UTQ48691.1 | D267N | - | D254N | - | *K. pneumoniae* |
| NDM-44 | UVJ50740.1 | D267N | - | - | - | *K. pneumoniae* |
| NDM-45 | UZC76856.1 | D130A, A233V | V88L | M154L | - | *E. coli* |
| NDM-46 | UZC76857.1 | - | V88L | M154L | A18V | *E. coli* |
| NDM-47 | UZC76858.1 | Q151L | - | - | - | *E. cloaceae* |
| NDM-48 | UZC76860.1 | - | V88L | P28H, M154L | - | N.A |
| NDM-49 | WAS27907.1 | - | V88L | M154L, P179R | - | *E. coli* |
| NDM-50 | EMM3083081.1 | D130N | - | M154L | M22I | *K. pneumoniae* |
| NDM-51 | WEG43790.1 | - | V88L | M154L, A252D | - | *E. coli* |
| NDM-52 | WEM34775.1 | D130A | - | - | - | *K. pneumoniae* |
| NDM-53 | WEG44271.1 | - | V88L | R32H, M154L | - | *E. coli* |
| NDM-54 | WEG44272.1 | - | - | R32L | - | *K. pneumoniae* |
| NDM-55 | WEY36504.1 | A99T | V88L | M154L | - | *E. coli* |
| NDM-56 | WGO19549.1 | K268T | N57T, L87R, V88L | M154L, K242Q | - | *E. coli* |
| NDM-57 | WGO19550.1 | - | V88L | M154L | E2G | *E. coli* |
| NDM-58 | WIF29698.1 | - | - | P185S | - | *P. aeruginosa* |
| NDM-59 | WIU89415.1 | D130N | - | E152K, M154L | - | *E. coli* |
| NDM-60 | WJL30768.1 | - | V88L | M154L, D202N | - | *E. coli* |
| NDM-61 | HDV0005713.1 | D130G, A233V | - | - | - | *K. pneumoniae* |
| NDM-63 | WOE87881.1 | - | ∆F70, A72H | G69S | - | *K. pneumoniae* |
| NDM-64 | WVS53334.1 | - | - | - | A20V | *P. aeruginosa* |
| NDM-65 | WVW91587.1 | G222S | - | - | - | *E. cloaceae* |
| NDM-66 | WXB24425.1 | - | I79L, V88L | M154L | - | *E. coli* |
| NDM-67 | WXH45424.1 | - | A72V, V88L | M154L | - | *E. coli* |
| NDM-68 | WXH45425.1 | - | V88L | I99L, M154L | - | *E. coli* |
| NDM-69 | BFJ38853.1 | - | - | - | E2K | *P. mirabilis* |
| NDM-70 | XBS36002.1 | - | V88L | M154L, G206A | - | *E. coli* |
| NDM-71 | XFH17879.1 | - | - | N110D | - | *K. pneumoniae* |
| NDM-72 | XFH17881.1 | - | V88L | M154L | S24N | *K. pneumoniae* |
| NDM-73 | XHO32899.1 | - | - | - | T15S | *K. pneumoniae* |
| NDM-74 | XHO32900.1 | D130A | - | E152K | - | *E. coli* |
| NDM-75 | XJP49379.1 | Q123L, D124H | V88L | M154L | - | *E. coli* |
| NDM-76 | XJP49380.1 | D130N | - | M154L | D43G | *C. koseri* |
| NDM-77 | XJP49381.1 | - | - | H12Q | - | *A. baumannii* |
| NDM-78 | XJP49382.1 | A238S | I46N, M48I, V88L | A52S, M154L | - | *E. coli* |
| NDM-79 | XLM53465.1 | D130A | - | M154L | - | *E. coli* |
| NDM-80 | XQU46182.1 | D130N | V88L | M154L | - | *E. coli* |
| NDM-81 | XLZ96866.1 | G222A | V88L | M154L | - | *E. coli* |
| NDM-82 | BFY84773.1 | - | - | - | M22I | *A. nosocomialis* |
| NDM-83 | XOU30558.1 | R64C | V88L | M154L | - | *E. coli* |
| NDM-84 | XPD12779.1 | - | R81M, V88L | M154L | - | *K. pneumoniae* |
| NDM-85 | XRI57706.1 | - | - | E152K, M154L | - | *E. coli* |
| NDM-86 | XRP76290.1 | - | A56T, V88L | M154L | - | *E. coli* |
| NDM-87 | XSG67055.1 | - | A72V | F70Y | - | *A. baumannii* |
| NDM-88 | MFL1403558.1 | - | V88L | M154L | M7I, S24G | *E. coli* |
| NDM-89 | XUZ45621.1 | - | - | Q123Y | - | *P. aeruginosa* |
| NDM-90 | XUZ93898.1 | - | V88L | M154L | E2A | *E. coli* |
| NDM-91 | XUZ93899.1 | - | G83D, V88L | M154L | - | *E. coli* |


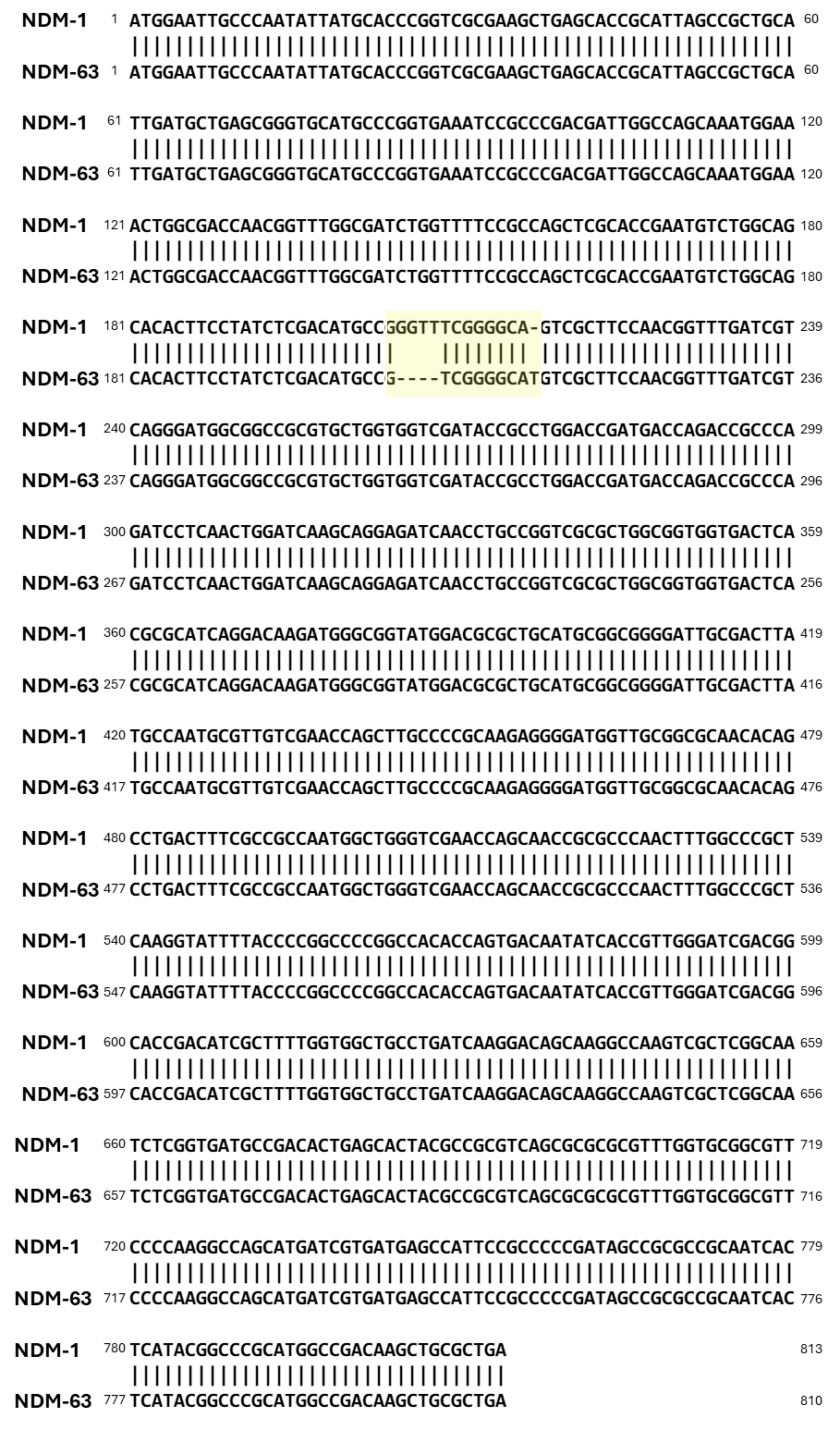
**Figure S1. Nucleotide alignment of NDM-63 in comparison with NDM-1 (Accession number CP071030.1).** Yellow shadow showed four-nucleotides deletions (∆GGTT) and one- nucleotide insertion (+T).


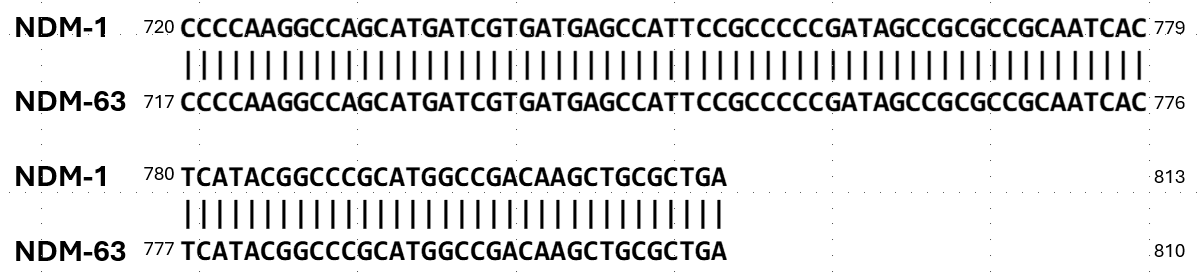


**Figure S2. Representative structures extracted from 500-ns MD simulations of (A) NDM-1 and (B) NDM-63.** Both proteins are shown in cartoon representation, with NDM-1 in orange and NDM-63 in cyan. Active-site residues are displayed in licorice representation, with C atoms in orange (NDM-1) or cyan (NDM-63), O atoms in red, S atoms in yellow, and N atoms in blue. Zinc ions are shown as gray spheres. C atoms of residues that differ between NDM-1 and NDM-63 are colored in pink.


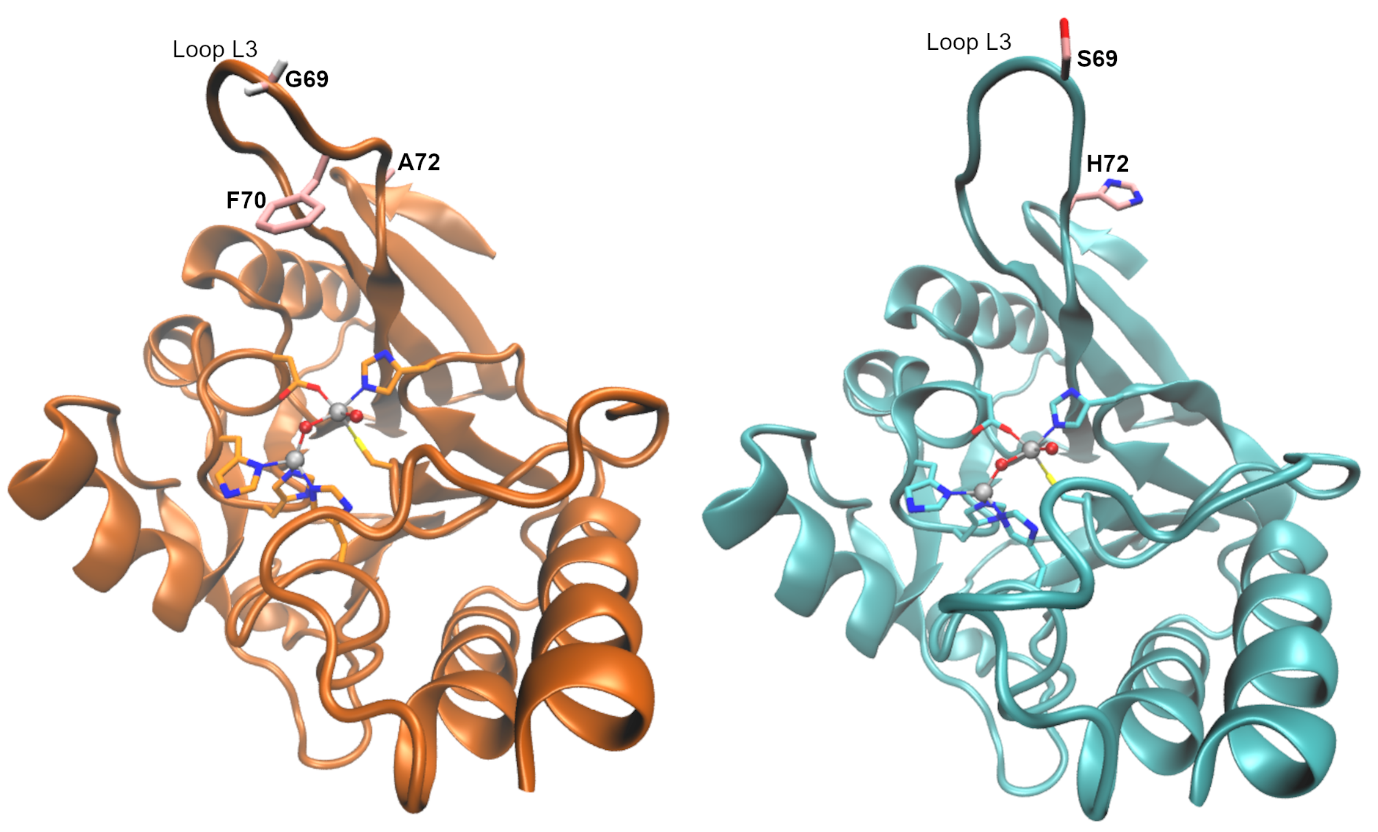

Supplement: Supplemental material — Tables S1 and S2; Fig. S1 and S2. [file aac.01286-25-s0001.docx]
